# Determinants of respiratory tract aerosol generation in a diverse clinical population: an observational study

**DOI:** 10.1136/bmjresp-2025-003494

**Published:** 2025-12-10

**Authors:** George W Nava, Alicja Szczepanska, Liji Ng, Sharzib Khan, Fergus Hamilton, Rachel Scott, Umair Mahmud, AERATOR group, D Arnold, Dinesh Saralaya, Nick Maskell, Jonathan P Reid, Bryan R Bzdek, James W Dodd

**Affiliations:** 1 Academic Respiratory Unit, University of Bristol, Bristol, UK; 2 NIHR Bristol Biomedical Research Centre, Bristol, UK; 3 North Bristol Lung Centre, NHS North Bristol NHS Trust, Bristol, UK; 4 School of Chemistry, University of Bristol, Bristol, UK; 5 Bradford Institute for Health Research, Bradford, UK; 6 Department of Infection Science, North Bristol NHS Trust, Bristol, UK

**Keywords:** Respiratory Infection, Infection Control

## Abstract

**Rationale:**

Respiratory tract infections are transmitted in part by infectious aerosol. Developing a greater understanding of how clinical and demographic factors affect aerosol generation could help to identify airborne infection ‘superspreaders’.

**Objectives:**

To measure respiratory aerosol from a diverse clinical population, exploring the impact of demographics, physiological factors and disease status.

**Methods:**

We recruited people with chronic lung disease, respiratory infection and healthy volunteers. We sampled aerosol from an enclosed circuit to exclude background non-respiratory aerosol, uniquely enabling bedside measurements of respiratory aerosol generation from an unwell population, while participants performed simple manoeuvres such as speaking and coughing.

**Measurements and main results:**

Across 128 participants, we detected lower aerosol generation among patients with a lung disease during a forced expiratory manoeuvre. This is likely to be related to differences in forced exhalation rather than demographic or clinical status. We observed a 500-fold variation in peak aerosol production when coughing. There was an association between aerosol generation and higher body mass index during coughing, but not with other clinical or demographic factors, and most of the variation remained unexplained.

**Conclusions:**

Our measurement of respiratory aerosol generation from patients with lung disease and infection is comparable with those published previously for healthy subjects. The amount of aerosol generation across the studied population was most closely linked with expiratory flow. While we observed variation in respiratory aerosol generation between participants in a clinical environment, there was no meaningful impact of demographics or respiratory disease on aerosol generation.

**WHAT IS ALREADY KNOWN ON THIS TOPIC**

- There is significant variation in aerosol production between different people which may influence the risk of infection transmission. Previous studies have identified relationships with factors such as age, body mass index (BMI) and underlying respiratory disease and aerosol generation; however, these findings are not always consistent, and much of the observed inter-person variation remains unexplained.

**WHAT THIS STUDY ADDS**

- Our findings suggest that the force of exhalation is the primary factor determining emitted aerosol concentration. We did not find a significant association between demographic or clinical characteristics and substantial differences in aerosol generation, indicating that these factors are unlikely to be key determinants of infection spread.

**HOW THIS STUDY MIGHT AFFECT RESEARCH, PRACTICE OR POLICY**

- These insights enhance our understanding of aerosol generation in the individual and will help inform infection control policies and future pandemic preparedness efforts.

## Introduction

 The spread of SARS-CoV-2 resulted in the deaths of over 7 million people.^1^ During the COVID-19 pandemic, concerns about airborne virus transmission due to respiratory tract aerosol generation were burdensome and restricted the delivery of healthcare services. Respiratory aerosols are an important mode of viral transmission that is not mitigated by contact infection control strategies such as handwashing.^2^ The development of effective and practical infection control policies designed to limit nosocomial spread of respiratory viruses requires a detailed understanding of the factors that determine the quantity of infection aerosol particle generation.

The use of the term ‘superspreader’ was popularised during the COVID-19 pandemic, though it was used in different ways to refer to physical or behavioural characteristics of individuals, groups or events.^3^ Some individuals produce higher number or mass concentrations of aerosol than others and as a result might become superspreaders were they to develop a respiratory infection. The demographic, clinical and physiological characteristics associated with increased aerosol generation have not been widely investigated. Furthermore, most aerosol generation studies have been conducted in healthy control participants, so it is unclear whether the aerosol-generating potential of a clinical population differs from that of the healthy control.

In this study, we collect aerosol originating from the respiratory tract while minimising the contribution of ambient aerosol, a contribution that has previously limited measurements from being taken at the patient’s bedside. This enables measures of aerosol generated by more relevant clinical populations including patients with chronic lung disease and patients with acute respiratory tract infections. We aim to examine whether patient-specific factors associate with greater respiratory tract aerosol generation.

## Methods

We identified participants from North Bristol NHS Trust and Bradford Teaching Hospitals NHS Foundation Trust, UK. Participants included healthy volunteers, patients with chronic disease and patients with acute respiratory illness from inpatient and outpatient areas. Acute respiratory illness included patients with a clinical diagnosis of respiratory infection or exacerbations of underlying airways disease.

We collected demographic and clinical information from participants. Participants performed respiratory manoeuvres while wearing an unvented non-invasive ventilation mask connected to aerosol measurement devices. This protocol included periods of tidal breathing, deep breathing, speaking, performing a forced spirometry manoeuvre and coughing (see online supplemental methods for full details).

We used two devices, an Optical Particle Sizer (TSI 3330, TSI, USA; particle diameters 0.3–10 µm) and an Aerodynamic Particle Sizer (TSI 3321, TSI, USA; particle diameters 0.5–20 µm) to sample the number concentration of aerosol in the mask. The devices draw in air at rates of 1 and 5 L min^−1^, respectively, and were set to report the aerosol number concentration at 1 s resolution. Particle transmission losses from the apparatus were characterised in previous work.^4 5^ Background aerosol number concentrations in the room were significantly higher than air drawn through the particle filter into the mask, which allowed us to identify and intervene if there was air leak due to an inadequate mask fit.

We analysed the data using the approach as previously described.^5^ A sample size of 25 was required to detect differences in aerosol generation of a factor of 2.5 with a power of 90% to detect differences, allowing for a dropout rate of 10%, as previously calculated.^4^ For breathing and speaking, we calculated the arithmetic mean of aerosol generation for the duration of the activity for each participant, while we calculated the arithmetic mean of the peak values of aerosol generation for each participant for forced vital capacity (FVC) manoeuvres and cough. We log-transformed positively skewed data. We compared aerosol number concentrations using two-tailed Student’s t-tests, two-way analyses of variance (ANOVA)^6^ and linear regression. A Benjamini-Hochberg 5% false discovery rate (FDR) was used to adjust p values between the six ANOVAs. We constructed a multiple linear regression model using explanatory variables that the investigators considered candidates for influencing aerosol generation. We designed each model based on the confounding structure illustrated in the directed acyclic graph (DAG) (see online supplemental figure E2). We conducted relevant sensitivity analyses such as including spontaneous cough outcomes of the multiple linear regression model and examining the relationships between age and body mass index (BMI) with tidal/deep breathing.

We estimated particle exhalation rates for each participant as done previously.^4^ The aerosol instrumentation provides a measure of the concentration of aerosol in the respiratory plume. The flow rate of the respiratory plume was measured from minute ventilation or FVC and typically was greater than the flow rate of the aerosol sampling setup (11 L/min). For tidal breathing, the particle exhalation rate was calculated from the product of the mean aerosol number concentration and the minute ventilation (MV), and for the FVC manoeuvre the product of the mean peak aerosol number concentration and the forced expiratory volume in 1 second(FEV1). We combined the two datasets and calculated a Pearson’s correlation coefficient. Statistical analyses were performed using Stata MP (V. 18). Patients and the public were not involved in the design, conduct or reporting of this study.

### Patient and public involvement

This work stemmed from the National Institute for Health and Care Research and Medical Research Council (UK Research and Innovation) Rapid Response call, part of the UK COVID pandemic response, the original studies included work with patients and healthy volunteers which informed the development of the sample techniques used in this study.

## Results

We recruited 38 healthy volunteers, 48 people with chronic lung disease and 42 people with acute respiratory tract illnesses (with and without underlying lung disease) (see table 1).

**Table 1:** Demographics

Characteristic	No lung disease, well	No lung disease, unwell	Lung disease, well	Lung disease, unwell	P value*
	n=38	n=11	n=48	n=31	
Sex n (%)					0.34
Male	11 (28.9)	6 (54.5)	21 (43.8)	11 (35.5)	
Female	27 (71.1)	5 (45.5)	27 (56.2)	20 (64.5)	
Age median (IQR)	35 (29–44.8)	61 (54–69.5)	56 (39.8–64)	70 (55.5–80)	<0.001
Ethnicity n (%)					0.67
White	29 (76.3)	7 (63.6)	35 (72.9)	27 (87.1)	
Asian	7 (18.4)	3 (27.3)	10 (20.8)	4 (12.9)	
Black	2 (5.3)	1 (9.1)	2 (4.2)	0	
Mixed	0	0	1 (2.1)	0	
Smoking history n (%)					0.001
Current	2 (5.3)	3 (27.3)	5 (10.4)	6 (19.4)	
Ex	7 (18.4)	3 (27.3)	26 (54.2)	15 (48.4)	
Never	29 (76.3)	5 (45.5)	17 (35.4)	10 (32.3)	
Underlying respiratory disease n (%)	–	–			
Asthma	–	–	30 (62.5)	9 (29.0)	
COPD	–	–	11 (22.9)	13 (41.9)	0.02
Asthma and COPD	–	–	4 (8.3)	2 (6.5)	
Bronchiectasis	–	–	2 (4.2)	5 (16.1)	
Interstitial lung disease	–	–	1 (2.1)	2 (6.5)	
Self-reported productive cough n (%)	0	5 (45.5)	9 (18.8)	24 (77.4)	<0.001
Missing	1 (2.6)	1 (9.1)	2 (4.2)		
Inpatient n (%)	4 (10.5)	10 (90.9)	0	26 (83.9)	<0.001
If unwell, length symptoms <8 days n (%)	–	7 (63.6)	–	12 (38.7)	0.18
If unwell, pathogen identified n (%)					0.46
Virus	–	3 (27.3)	–	7 (22.6)	
Bacteria	–	2 (18.2)	–	6 (19.4)	
Mycobacteria	–	1 (9.1)	–	0	
No positive sample	–	5 (45.5)	–	18 (58.1)	
BMI (kg/m^2^) median (IQR)	26.1 (22.9–29.0)	29.5 (27.3–32.6)	27.9 (23.7–32)	27.3 (22.9–32.8)	0.16
FEV1 (% predicted) mean (SD)	89 (18.8)	56.3 (16.3)	69.5 (25.2)	53.7 (20.2)	<0.001
Missing n (%)	1 (2.6)	2 (18.2)	1 (2.1)	2 (6.5)	
FVC (% predicted) mean (SD)	92.4 (17.4)	60.3 (20.2)	83.4 (22.6)	64.3 (19.4)	<0.001
Missing n (%)	1 (2.6)	2 (18.2)	1 (2.1)	2 (6.5)	
Minute ventilation (L/min) mean (SD)	10.3 (3.33)	13.5 (2.84)	12.6 (3.38)	13.9 (3.62)	0.02
Missing n (%)	22 (57.9)	4 (36.4)	13 (27.1)	17 (54.8)	

* Fisher’s exact test performed for categorical variables, and Kruskal-Wallis or one-way ANOVA for continuous variables dependent on whether the data were normally distributed.

BMI, body mass index; COPD, chronic obstructive pulmonary disease; FEV1, forced expiratory volume in 1 second; FVC, forced vital capacity.

Broadly, our measurements of aerosol number concentrations across the participant groups and between respiratory manoeuvres are comparable with those published previously for healthy subjects,^4 7 8^ suggesting that the variations between the different participant groups are only minor at most. These previous studies had all been undertaken in ultraclean laminar flow environments to ensure unambiguous identification and quantification of exhaled aerosol particles above the high background ambient aerosol levels present in most rooms (see online supplemental figure E3).

**Figure 1:**
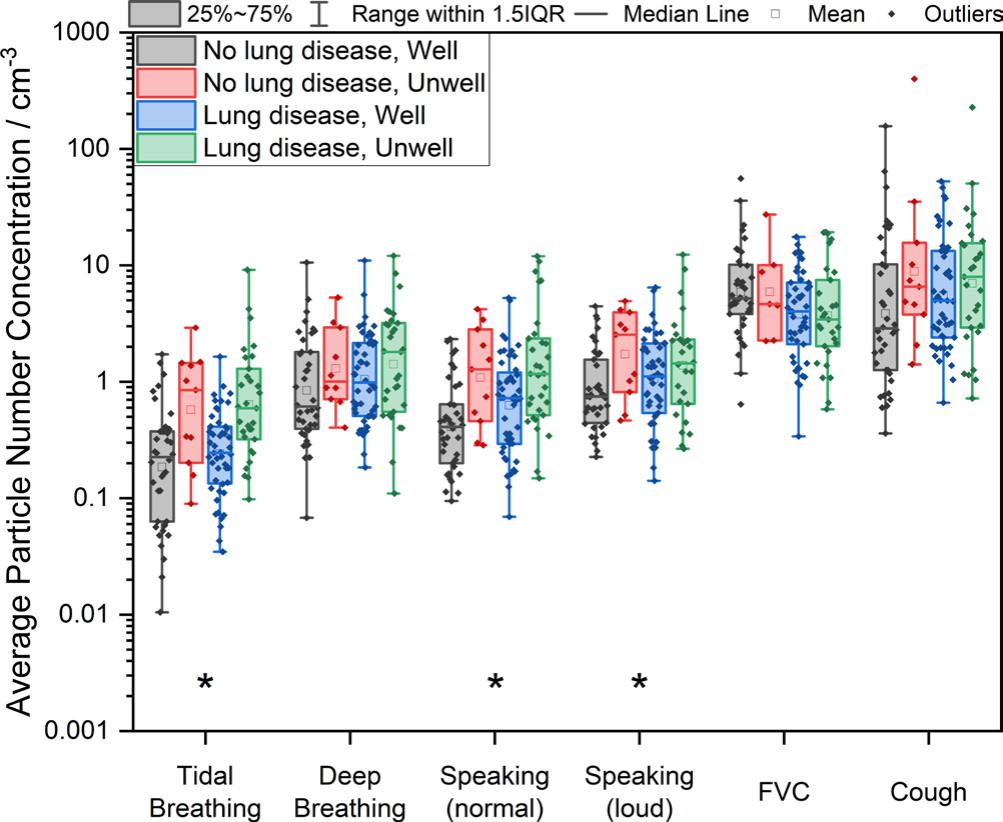
Exhaled air aerosol number concentration (particles/cm^3^) during different respiratory manoeuvres. Participants grouped by the presence of chronic lung disease (lung disease or no lung disease) and acute respiratory illness (well or unwell). *Indicates a between-subject two-way analysis of variance p<0.05 after FDR adjustment. FVC, forced vital capacity; FDR, false discovery rate; IQR, interquartile range.

We show aerosol production to be highest during the cough and a forced exhalation manoeuvre across all participants (see figure 1). Having a chronic respiratory disease may be associated with lower aerosol number concentration during a forced expiratory manoeuvre (p=0.038); however, the significance of this is uncertain after accounting for multiple testing (FDR adjusted p=0.226). Similarly, there is evidence that having an acute respiratory illness is associated with higher aerosol generation with a voluntary cough (p=0.051, FDR adjusted p=0.076) (see online supplemental table E1). For the low airflow manoeuvres (tidal breathing and speaking at normal conversational volume), the analysis is more complex. These activities, particularly tidal breathing, produce very low concentrations of aerosol. A correlation between the background aerosol level and aerosol number concentration measured during tidal breathing (see online supplemental figure E4) suggests that these data may contain a component from the background air and must be interpreted with caution. A correlation, although much weaker, is observed between the background aerosol number concentration and that measured from normal speaking. However, a between-subject two-way ANOVA of each respiratory manoeuvre suggests that an acute respiratory illness may be associated with higher aerosol number concentration during tidal breathing (p<0.001) and normal speech (p<0.001).

Selecting cough as the most clinically significant respiratory manoeuvre, we have constructed a regression model using clinical and demographic variables based on our hypotheses that each factor might influence aerosol production. We found weak evidence of an association between higher BMI and the presence of a productive cough with higher aerosol number concentrations in both the unadjusted and adjusted models (see table 2). The models explained little of the overall variance in the dataset (R^2^ and adjusted R^2^<0.1 for all models).

**Table 2:** Univariable and adjusted linear regression models of peak aerosol number concentration during a voluntary cough (arithmetic log-mean of three individual coughs)

Explanatory variable	Unadjusted		Adjusted models	
	Beta coefficient and 95% CI	P value	Beta coefficient and 95% CI	P value
Age	0.01 (−0.00 to 0.02)	0.16		
Female	−0.36 (−0.84 to 0.12)	0.14		
BMI	**0.04 (0.01 to 0.08)**	**0.02**	**0.05 (0.01 to 0.09)**	**0.02**
Chronic lung disease	0.27 (−0.21 to 0.74)	0.27	0.16 (−0.35 to 0.67)	0.53
Acute respiratory illness	0.46 (−0.03 to 0.96)	0.07	0.32 (−0.24 to 0.88)	0.27
Productive cough	**0.65 (0.14 to 1.16)**	**0.01**	0.57 (−0.12 to 1.25)	0.10
FEV1	−0.15 (−0.38 to 0.08)	0.20	−0.04 (−0.37 to 0.30)	0.83

The multivariable model reports the association for each explanatory variable adjusted as informed by the DAG (online supplemental figure E2). The R2 and adjusted R2 of all univariable and multivariable models, respectively, are <0.1.

Bold values indicate statistical significance.

BMI, body mass index; DAG, directed acyclic graph; FEV1, forced expiratory volume in 1 second.

**Figure 2:**
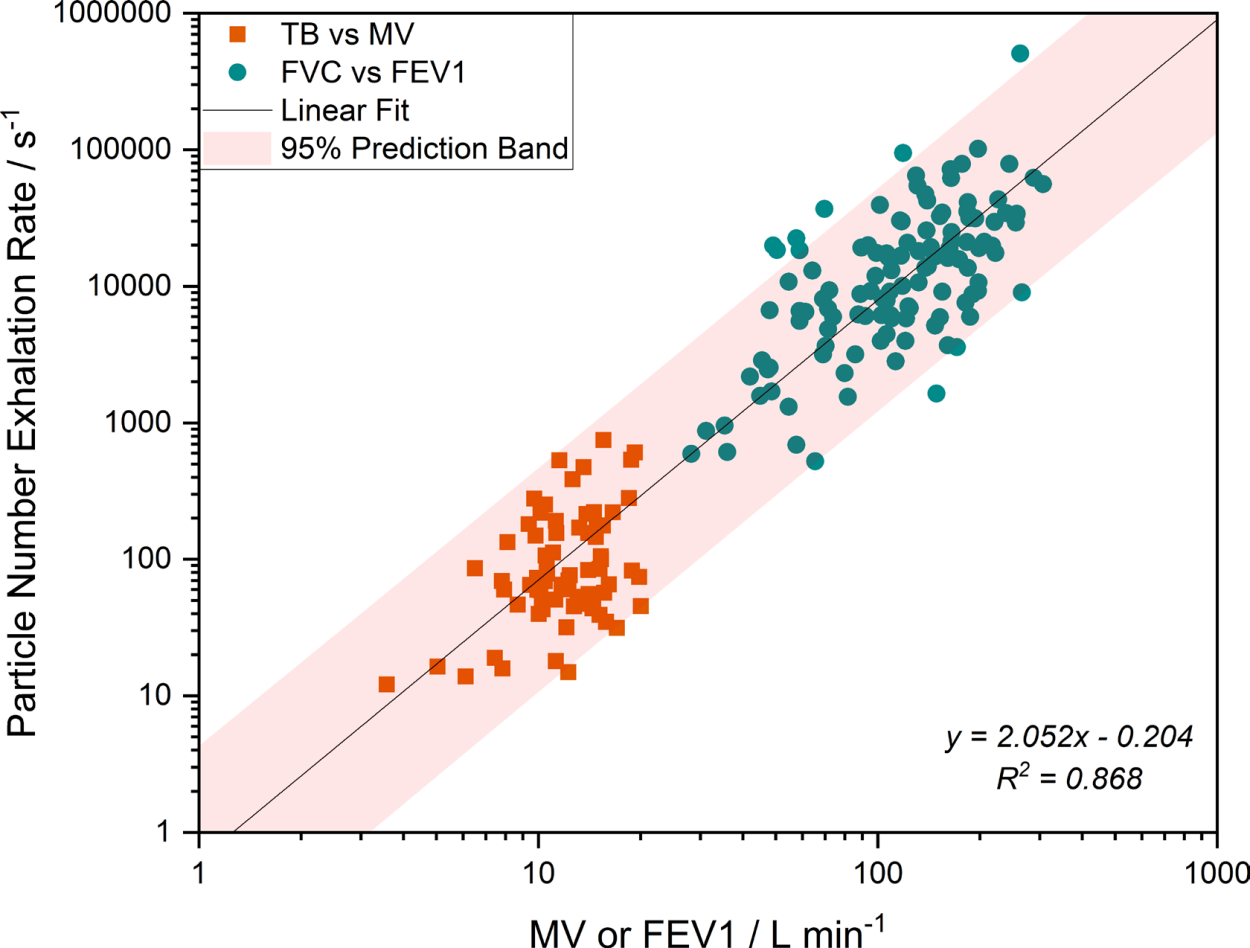
Particle number exhalation rate measured during TB and FVC plotted against MV and FEV1, respectively. The linear fit, its 95% prediction band, the linear fit equation and R² are shown. FEV1, forced expiratory volume in 1 second; FVC, forced vital capacity; MV, minute ventilation; R², coefficient of determination; TB, tidal breathing.

The absolute particle exhalation rate is calculated by multiplying the aerosol number concentrations (particles/cm^3^) produced during tidal breathing and during a forced exhalation manoeuvre by MV and FEV1, respectively. When compared with the exhalation rate (estimated from the minute ventilation and FEV1 measurements), we report a strong correlation (Pearson correlation coefficient=0.93), with an increase in aerosol generation with increase in airflow (see figure 2), highlighting the importance of the rate of airflow in the generation of aerosol.

## Discussion

To date, this is the largest study to measure respiratory aerosol generation in patients with acute and chronic respiratory disease. While there is some indication of marginally lower aerosol production with chronic respiratory disease during a forced exhalation and higher aerosol production with higher BMI during cough, the impact of these factors is likely minimal. However, there is a strong relationship between the force of a manoeuvre and aerosol generation. This is consistent with previous studies of aerosol production at rest, speaking and during exercise,^9 10^ indicating that overall, whether it is healthy participants or those with pre-existing lung conditions, infected or not, the main determinant of the aerosol yield appears to be how forcefully someone is exhaling.

Three important mechanisms are thought to influence respiratory aerosol production: (1) bronchiolar fluid film bursts during inhalation as collapsed terminal airways open, (2) shear forces along the mucus lined airways from turbulent flow and vocal cord vibrations and (3) the production of large droplets from saliva in the oral cavity during coughing.^11^ The forced expiratory manoeuvres likely increase aerosol generation through the first two mechanisms, while all three mechanisms are relevant to coughing. We show a relationship between the rate of air exhalation and the rate of aerosol generation when tidal breathing and with the FVC manoeuvre. This is consistent with previous work in healthy volunteers^9 10^ and in a small study of participants with rhinovirus.^12^ A reduction in aerosol number concentration in those with underlying respiratory disease during forced manoeuvre may be explained through reduced airflow due to their condition. This has been previously shown in a study comparing healthy volunteers and patients with chronic obstructive pulmonary disease (COPD),^13^ though not by Schwarz *et al*^14^ who found no relationship of aerosol emission with lung disease or airflow obstruction. Differences in findings could be explained by relatively small sample sizes and limitations of aerosol sampling methodology. In our study, the presence of an acute respiratory illness appears to be associated with an increase in aerosol number concentration during tidal breathing, speaking and potentially when coughing, but not with the forced manoeuvre. This is consistent with higher tidal breathing aerosol emission found in patients infected with COVID-19.^15 16^ This might relate to infection-associated hypersecretion of mucus leading to increased aerosol generation.^17^ However, our results must be interpreted with caution, since the background aerosol measurements correlate with measurements during low flow activities. Incomplete mitigation of the higher background aerosol concentration in the rooms of those with acute illness might explain the observed relationship during tidal breathing and speaking.

We have assessed the influence of a range of demographic, physiological and clinical factors that we hypothesised could be associated with aerosol production by altering airflow dynamics. Our analysis identifies that a higher BMI contributed modestly to peak aerosol number concentration when coughing. This may be explained by increased atelectasis in individuals with a high BMI, leading to increased aerosol generation through bronchioalveolar film rupture as collapsed alveoli and terminal bronchi open. We also show that a self-reported productive cough is associated with increased aerosol generation, though this is not evident once adjusted for the presence of underlying respiratory disease and acute illness. Our model is only able to explain a small proportion of the variance seen within our cough data; therefore, it is unlikely that these factors would be of clinical use to identify those who might be most infective.

Other studies have investigated the relationship between aerosol generation and demographics, though these results are not always consistent. Our findings align with those of Archer *et al*, who found no difference in exhaled air particle number concentration during tidal breathing in relation to age of participants from 12 to 72.^10^ However, they contrast with those of Schumm *et al*^18^ who examined aerosol emission in healthy volunteers at rest and with exercise. They compared people aged 20–39 with people aged 60–76 and found that aerosol emissions increased with age though did not change with BMI. Our data may differ from that of Schumm *et al* due to our population with participants aged 22–90 years and BMI ranging from 16.0 to 45.9 kg/m^2^, compared with their age range of 12–72 and BMIs of 18.0–33.3 kg/m^2^. It might also relate to the manoeuvre that is analysed, since a regression of our data for tidal breathing and deep breathing (rather than coughing) suggests older participants emit higher number concentrations of aerosol and BMI is not associated with a difference (see online supplemental tables E2 and E3). Bake *et al*^19^ assessed a large group of volunteers and reported age, weight and spirometric values to account for a considerable amount of the inter-person variation (28–29%). However, comparison with our data is limited by their population being restricted to healthy middle-aged individuals, and they analysed aerosol generation during a ‘standardised breathing manoeuvre’ rather than our assessment of a real-world aerosol-generating cough. Another study^16^ found a small effect of BMI on aerosol generation in healthy volunteers during tidal breathing, though no association has been found between aerosol generation and BMI when speaking and coughing.^20 21^ Both latter studies were performed in healthy volunteers with BMI ranges much narrower than in our study.

There are multiple strengths of this study. We have collected aerosol emissions from a large diverse study population in two different hospitals in multiple settings, which increases the generalisability of our findings. We used a robust aerosol sampling methodology that allowed us to collect data outside of an ultraclean laminar flow environment. As a result, we were able to include patients who would previously have been excluded due to infection control concerns. Furthermore, we measured aerosol simultaneously with two different aerosol detection systems, which means we can validate the accuracy of our recordings.

There are also limitations to the study. First, we measure the number concentration of aerosol emitted on the assumption that infectious disease is more readily spread when a greater volume of aerosol is produced. This highlights the importance of measuring the biological content of aerosol to assess infectivity. However, there remain significant challenges in identifying which droplet sizes contain pathogens and quantifying directly the amount of pathogen on exhalation. In the absence of robust methods to achieve this, measurements of aerosol yield must frequently be used as the best indicator of transmission risk, although with caveats. Second, our measurement of ventilation and airflow was taken at different times to the measurement of aerosols; the high flow rates of the aerosol measurement devices can be expected to modify the recorded flow rates in a complex manner. Third, the real-world nature of this study can be seen both as a strength and a limitation, with the possibility of introducing unmeasured confounding variables (such as ambient temperature and humidity) when recording aerosols in different environments. Furthermore, different clinical environments had different background concentrations, which can be partly (but not wholly) mitigated by the filtration setup in our measures. Fourth, we used aerosol generation during voluntary cough as our primary analysis since we believe it to be of most clinical significance. It is possible that spontaneous coughing due to a respiratory tract illness might have a different aerosol profile and yield differing results. An analysis of data that includes spontaneous coughs of patients captured ad hoc during the experiment produced the same outputs to the regression analyses (see online supplemental table E4), which makes us more confident that our findings are robust. Finally, we capture aerosol generation as a single timepoint in the individual’s illness, rather than taking repeated longitudinal measures. It is possible that aerosol emission does increase during an acute illness, but only within a limited timeframe and by a magnitude that is too small to be detected among the large interparticipant variability in this study.

### Impact of our findings

Our previous work demonstrated that the most important factor in aerosol generation is not the medical procedure being undertaken at the time.^22^ Our study confirms a large interindividual variation in aerosol generation in a diverse population of patients. Our data do not provide compelling evidence to support that differences in patient-specific demographic or clinical status significantly impact risk of generating potentially infectious aerosols. Infection control policies should focus on the broader aspects of transmission, which include proximity, duration of exposure and the environment, rather than the procedure or specific clinical characteristics. This will help inform infection control policies for future pandemic preparedness.

## Conclusions and future work

Variation in respiratory tract aerosol generation in this study could not be explained by patient demographics and clinical characteristics. However, aerosol generation increased exponentially with increased airflow during exhalation, making it a key determinant of aerosol yield. Infection control strategies should consider focusing on the force of exhalation rather than patient demographic or clinical characteristics. Further work is required to establish aerosol generation at low flow respiratory activities such as speaking, and to assess biological content of exhaled breath to better understand the aerosol exposure risk required to transmit infection.

## Supplementary material

10.1136/bmjresp-2025-003494 online supplemental file 1

## Data Availability

Data are available upon reasonable request.
