# Supplementary material for: Determinants of respiratory tract aerosol generation in a diverse clinical population: an observational study
Source: BMJ Open Respir Res. 2025 Dec 10;12(1):e003494. doi: 10.1136/bmjresp-2025-003494 (PMC12699571; doi:10.1136/bmjresp-2025-003494)
Supplement: online supplemental file 1 [file bmjresp-12-1-s001.docx]

Online Data Supplement

**The determinants of respiratory tract aerosol generation in a diverse clinical population, an observational study.**

George W Nava^†, 1^, Alicja Szczepanska^†, 2^, Liji Ng^3^, Sharzib Khan^3^, Rachel Scott^1^, Fergus Hamilton^1,4^, Umair Mahmud^4^, AERATOR group, Dinesh Saralaya^4^, Jonathan P Reid^2^, Nick A Maskell^1^, Bryan R Bzdek^2^, James W Dodd^1^

^†^ These authors contributed equally to this work

**Supplementary Methods:**

Participants wore an unvented non-invasive ventilation mask (FreeMotion RT041 mask, Fisher&Paykel Healthcare, New Zealand; BiTrac NIV mask, Intersurgical Ltd, UK) with a particle filter (Flo-Guard Breathing Filter, Intersurgical Ltd, UK) through which all inhaled air passed (Supplementary figure E1 A. and B.). When using the Intersurgical masks, we removed anti-asphyxiation valves and sealed the exhalation port. We attached aerosol-sampling equipment to mask ports using conductive silicone tubing, 1.2 m in length (TSI 3001788, 4.83-mm inner diameter, 9.53-mm outer diameter) and 3D-printed connections made of polylactic acid (1.75 mm filament) (RAISE3D Pro2 Printer, 3DGBIRE, UK).

Participants completed a standardised protocol of two minutes of tidal breathing, three periods of deep breathing (two slow deep breaths over a 10-second period), three 15-second periods of speaking (the alphabet in a normal speaking voice (approximately 50 – 60 dB) at a rate of one letter per second; repeated with a loud speaking voice (approximately 70 – 80 dB), three forced expiratory manoeuvres (using a Forced Vital Capacity (FVC) manoeuvre) and three voluntary coughs. We controlled voice loudness with sound level meter (RS PRO RS-8852) and measured spirometry and minute ventilation (MV), with the aerosol-sampling devices detached from the mask with the spirometer (Bristol: Vyntus Spiro PC, Vyaire Medical Products Ltd, UK; Bradford: Pneumotrac Spirometer, Vitalograph Ltd, UK) attached to the particle filter through which all exhaled air travelled. We timestamped the recording when a patient spontaneously coughed.

**Figure E1.** A) An example of the arrangement of the non-invasive ventilation mask with particle filter attached. The black silicone tube attached to the oxygen ports allowed sampling of air within the mask by three machines simultaneously. When using the Intersurgical mask (such and in A)), the anti-asphyxiation valve was removed to allow exhalation through the particle filter, in addition the exhalation port in the mask was sealed to prevent unfiltered air from being inhaled. B) The arrangement of the aerosol sampling machines. Permission was obtained from authors featured in the images.


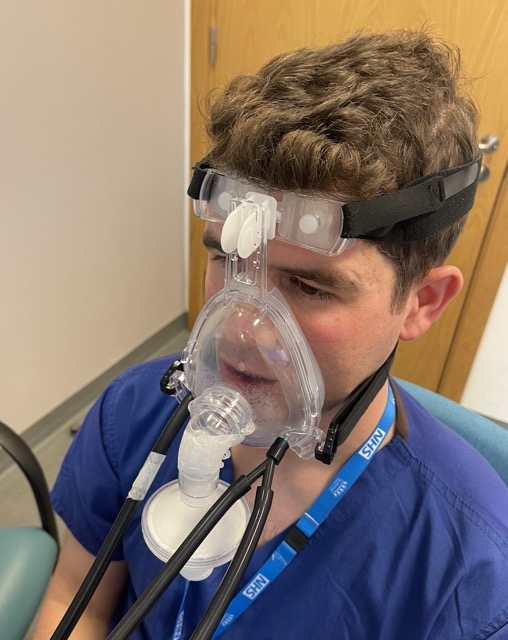
A)


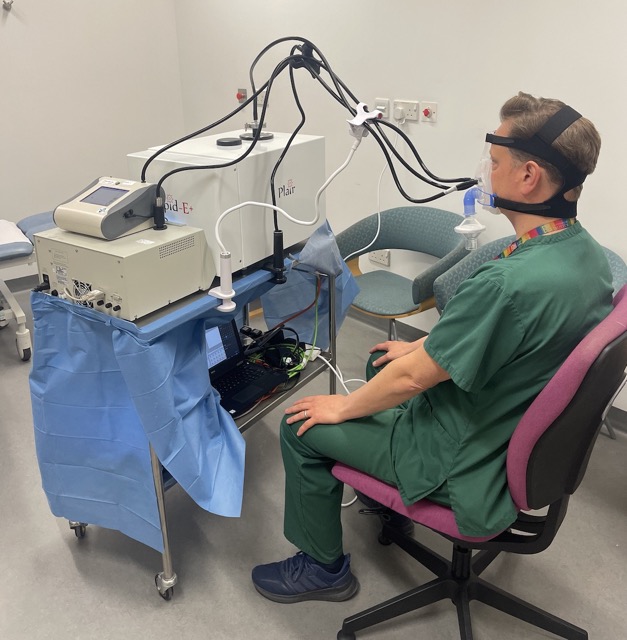
B)

**Figure E2– Directed acyclic graph describing the causal direction between demographic and clinical variables included in the regression models.**

**
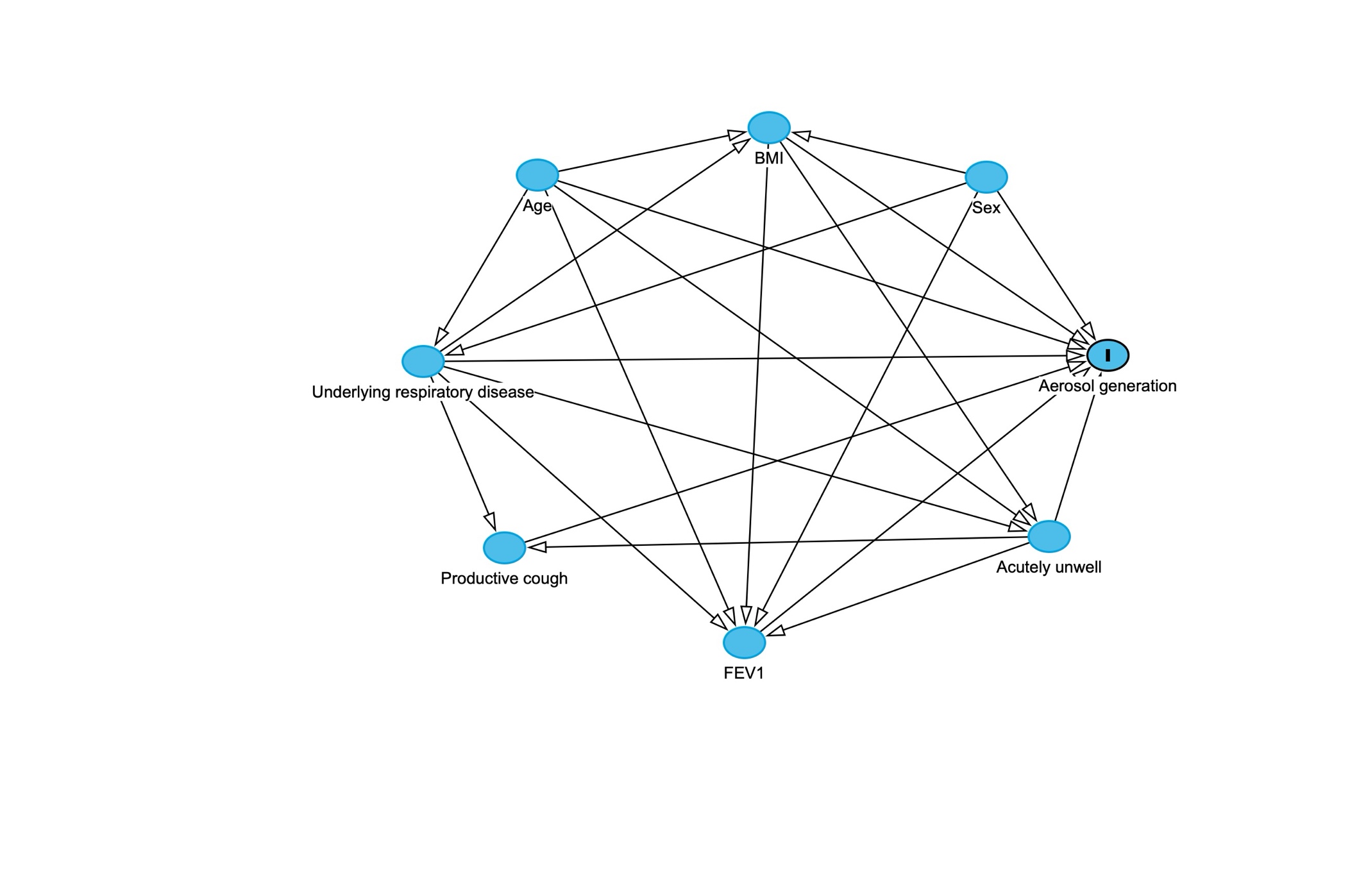
**

**Figure E3**. Comparison of our data with those reported in previous AERATOR and PERFORM (The Investigation of ParticulatE Respiratory Matter Release During performance and Exercise to inform Guidance in the SARS-CoV-2 PandeMic) publications(1-3) which collected aerosols from participants breathing into a funnel and will wearing an unfiltered cardiopulmonary exercise testing mask in an ultraclean laminar flow theatre.


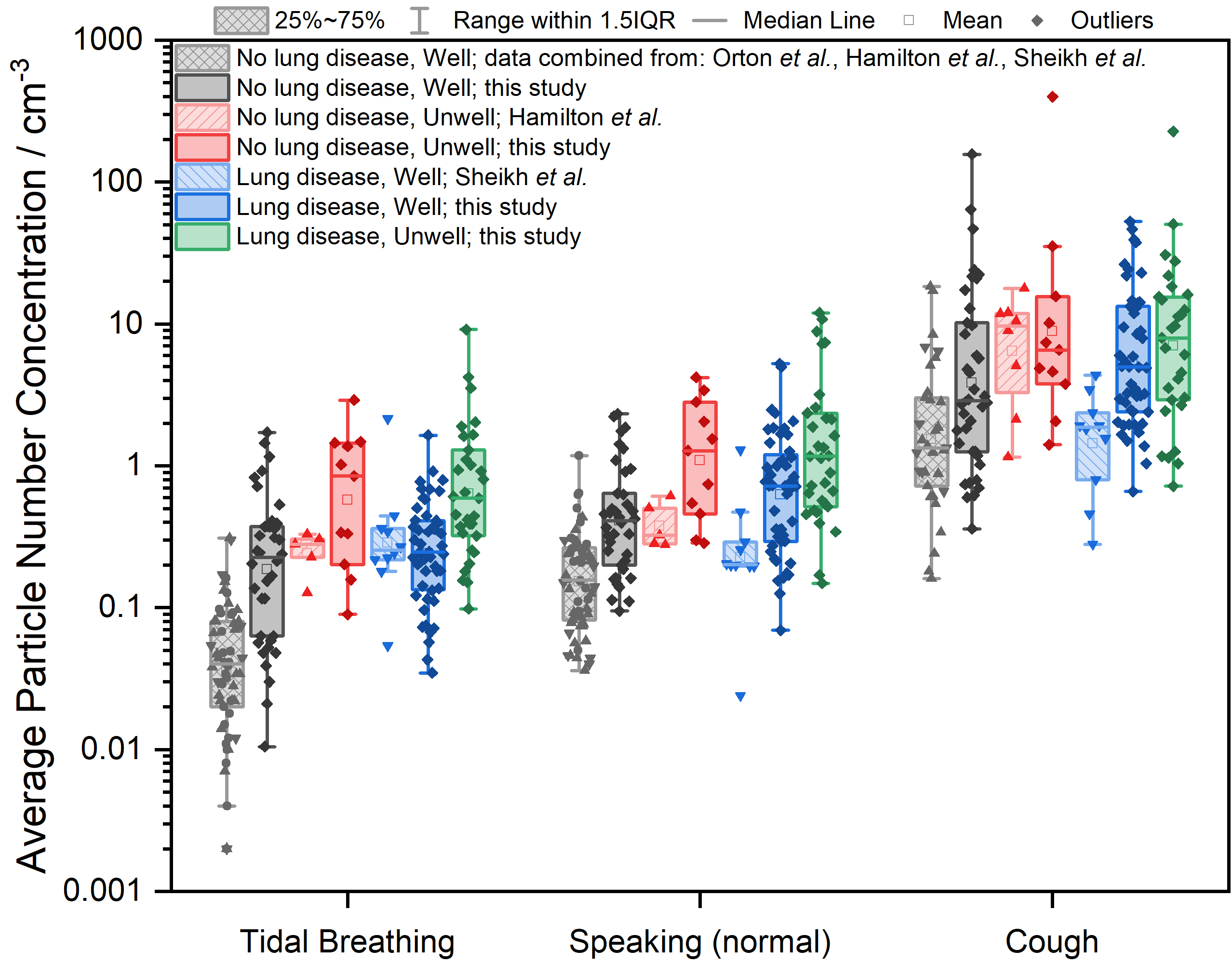


**Figure E4:** Scatter plots of the mean particle number concentration of the background air for each participant against the aerosol number concentrations measured during the respiratory manoeuvres: a) Tidal breath, b) Deep breathing, c) Speaking at a normal volume, d) Speaking loudly, e) An FVC manoeuvre, and f) Voluntary coughing.

1.
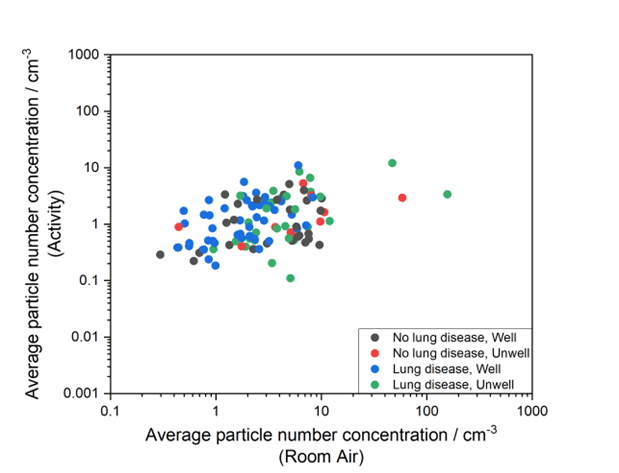

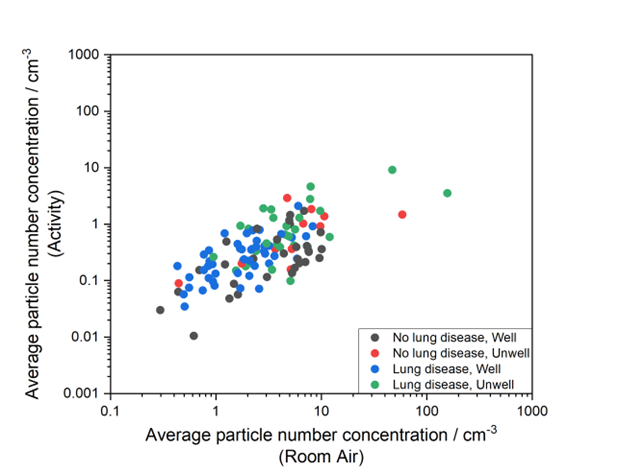
Tidal breathing b) Deep breathing
2. **
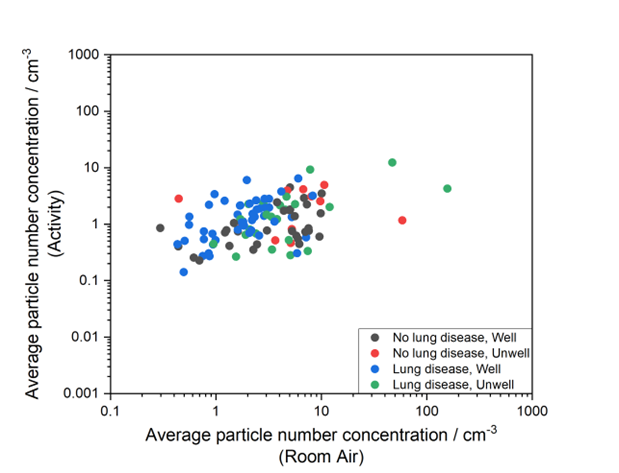

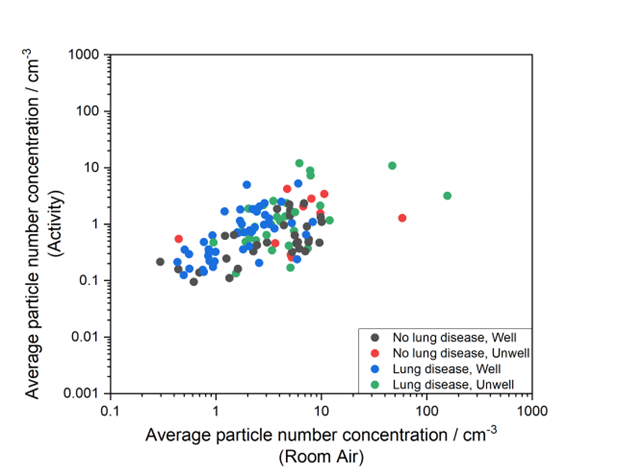
**Speaking at a normal volume d) Loud speaking

**
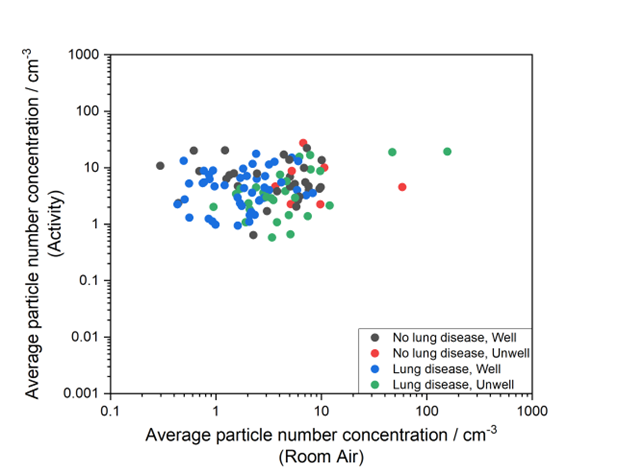

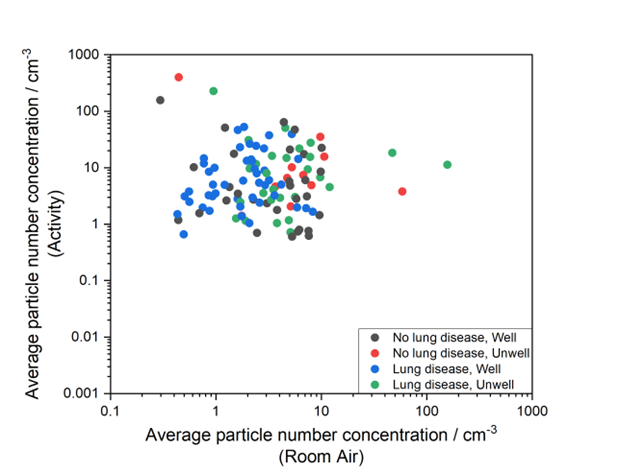
** e) FVC manoeuvre f) Voluntary coughing

**Table E1:** Between subject two-way ANOVA for each manoeuvre comparing analysing by the presence of acute illness (Unwell) and chronic lung disease. Adjusted P-values are reported after adjustment for multiple testing (5% FDR).

| Dependent variable | Exposure | F-ratio | P-value | Adjusted P-value |
| --- | --- | --- | --- | --- |
| Tidal breathing (mean aerosol number concentration) | Unwell | 24.1 | **<0.001*** | **<0.001*** |
|  | Chronic lung disease | 0.71 | 0.400 | 0.707 |
|  | Interaction | 0.14 | 0.708 | 0.898 |
| Deep breathing (mean aerosol number concentration) | Unwell | 3.04 | 0.084 | 0.101 |
|  | Chronic lung disease | 0.52 | 0.472 | 0.707 |
|  | Interaction | 0.10 | 0.748 | 0.898 |
| Normal speech (mean aerosol number concentration) | Unwell | 16.4 | **<0.001*** | **<0.001*** |
|  | Chronic lung disease | 1.87 | 0.174 | 0.522 |
|  | Interaction | 0.49 | 0.485 | 0.898 |
| Loud speech (mean aerosol number concentration) | Unwell | 7.38 | **0.008*** | **0.015*** |
|  | Chronic lung disease | 0.01 | 0.937 | 0.937 |
|  | Interaction | 1.63 | 0.204 | 0.816 |
| FVC manoeuvre (mean of the peak aerosol number concentrations) | Unwell | 0.00 | 0.959 | 0.959 |
|  | Chronic lung disease | 4.42 | **0.038*** | 0.226 |
|  | Interaction | 0.00 | 0.945 | 0.945 |
| Cough (mean of the peak aerosol number concentrations) | Unwell | 3.89 | 0.051 | 0.076 |
|  | Chronic lung disease | 0.06 | 0.814 | 0.937 |
|  | Interaction | 1.22 | 0.272 | 0.816 |

*degrees of freedom = 1 for all reported effects

**Table E2.** Regression analyses of aerosol generation on age. A) mean aerosol number concentration during tidal breathing and B) mean aerosol number concentration during deep breathing. A univariable analysis was performed followed by a multivariable analysis adjusting for other demographic, clinical and physiological factors (sex, ethnicity, BMI, chronic lung disease, acute respiratory infection, productive cough and FEV1).

A) Tidal breathing

|  | | **Unadjusted** |  | **Adjusted model** |  |
| --- | --- | --- | --- | --- | --- |
| **Explanatory**  **variable** | **Beta coefficient and 95% confidence interval** | | **P-value** | **Beta coefficient and 95% confidence interval** | **P-value** |
| Age | .02 (.01, .03) | | <.001 | .01 (-.00, .03) | .154 |

B) Deep breathing

|  | | **Unadjusted** |  | **Adjusted model** |  |
| --- | --- | --- | --- | --- | --- |
| **Explanatory**  **variable** | **Beta coefficient and 95% confidence interval** | | **P-value** | **Beta coefficient and 95% confidence interval** | **P-value** |
| Age | .02 (.01, .03) | | <.001 | .03 (-.01, .04) | <.001 |

**Table E3.** Regression analyses of aerosol generation on BMI. A) mean aerosol number concentration during tidal breathing and B) mean aerosol number concentration during deep breathing. A univariable analysis was performed followed by a multivariable analysis adjusting for other demographic, clinical and physiological factors (age, sex, ethnicity, chronic lung disease, acute respiratory infection, productive cough and FEV1).

A) Tidal breathing

|  | | **Unadjusted** |  | **Adjusted model** |  |
| --- | --- | --- | --- | --- | --- |
| **Explanatory**  **variable** | **Beta coefficient and 95% confidence interval** | | **P-value** | **Beta coefficient and 95% confidence interval** | **P-value** |
| BMI | .03 (-.01, .06) | | .101 | .02 (-.01, .05) | .142 |

B) Deep breathing

|  | | **Unadjusted** |  | **Adjusted model** |  |
| --- | --- | --- | --- | --- | --- |
| **Explanatory**  **variable** | **Beta coefficient and 95% confidence interval** | | **P-value** | **Beta coefficient and 95% confidence interval** | **P-value** |
| BMI | .02 (-.01, .05) | | .117 | .02 (-.01, .05) | .136 |

**Table E4.** A univariable and multivariable linear regression models of peak aerosol number concentration during all coughing episodes that participants had during record (log-mean of three voluntary coughs in addition to spontaneous coughs that occurred during the protocol). The multivariable model reports the association for each explanatory variable once adjusted for each of the other variables.

|  | | **Unadjusted** |  | **Adjusted model** |  |
| --- | --- | --- | --- | --- | --- |
| **Explanatory**  **variable** | **Beta coefficient and 95% confidence interval** | | **P-value** | **Beta coefficient and 95% confidence interval** | **P-value** |
| Age | 0.01 (-0.00, 0.02) | | 0.107 |  |  |
| Female | -0.33 (-.079, 0.13) | | 0.160 |  |  |
| BMI | **0.04 (0.00, 0.08)** | | **0.030*** | **0.04 (0.01, 0.08)** | **0.02*** |
| Chronic lung disease | 0.29 (-0.17, 0.75) | | 0.216 | 0.17 (-0.32, 0.67) | 0.49 |
| Acute respiratory illness | 0.45 (-0.03, 0.93) | | 0.064 | 0.29 (-0.26, 0.83) | 0.30 |
| Productive cough | **0.63 (0.14, 1.13)** | | **0.013*** | 0.53 (-0.13, 1.20) | 0.12 |
| FEV1 | -0.13 (-0.35, 0.09) | | 0.202 | 0.02 (-0.29, 0.34) | 0.89 |

*results with a statistical significance <0.05
